# Modeling of an air-to-air exchanger with dual-core in cascade connection

**DOI:** 10.1016/j.mex.2021.101253

**Published:** 2021-01-26

**Authors:** Haïfa Souifi, Yassine Bouslimani, Mohsen Ghribi, Serge Colin

**Affiliations:** a Electrical Engineering Department, University of Moncton, NB, Canada; b ClairiTech Innovations Inc, Boudreau-Ouest, NB, Canada

**Keywords:** Hybrid-core, Heat/energy recovery, Mathematical model, Effectiveness, Sensible heat transfer, Latent heat transfer

## Abstract

With the current leaning towards finding effective solutions to maintain a good indoor air quality (IAQ) inside houses and buildings and to simultaneously reduce the energy consumption, air-to-air exchangers with heat/energy recovery have emerged as one of the promising technologies to provide a healthy and comfortable indoor environment. To deeply evaluate these systems performances, the present investigation focuses on modeling a combined ERV-HRV exchanger using the effectiveness-NTU (ε−NTU) method. To this end, a detailed mathematical model introducing heat and mass exchange mechanisms was developed and applied to predict the system energy recovery efficiency. To assess its suitability, the developed model was validated and compared to real measurements which carried out under the Atlantic Canada weather. The comparison findings disclosed that the developed model can predict the system performance with a maximum relative discrepancy less than 10%.

• The detailed mathematical model including heat and mass exchange mechanisms was clearly developed using the ε−NTU approach in order to carefully predict the dual-core system performance in terms of sensible and latent recovery potential.

• The developed model was validated against real data to evaluate its suitability and accuracy. Obtained results show that it could be satisfactory for predicting the dual-core system performances.

• The ε−NTU approach adopted in this study could be a convenient method for modeling single or/and dual-core air-to-air heat/energy recovery systems.

## Graphical abstract

**Figure:**
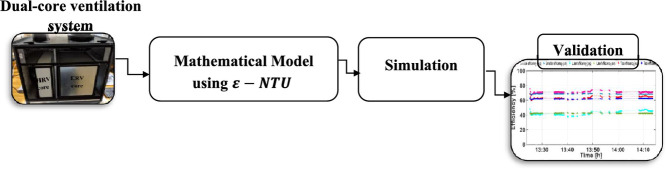

Specifications table

**Table:** 

Subject Area	*Engineering*
More specific subject area	*Mechanical ventilation systems*
Method name	*effectiveness-NTU (i.e.*ε−NTU*) method*
Name and reference of original method	G. Zhou et al., Modeling air-to-air plate-fin heat exchanger without dehumidification, Applied Thermal Engineering 143 (2018) 137-148, https://doi.org/10.1016/j.applthermaleng.2018.07.064
Resource availability	There are no special resources.

## Method details

### Background

According to many research studies [1–8], ensuring ventilation, via air-to-air heat/energy recovery systems (HRV/ERV) could provide healthy and comfortable indoor environment. By recovering heat/humidity between fresh and exhaust airstreams, HRVs/ERVs are widely adopted to significantly reduce the cooling and heating costs [1–8]. Thus, these ventilation systems are becoming considerably used worldwide, particularly in cold or humid weather regions [1,^2^,^7^,8]. Although several kinds of heat/energy recovery systems are available, the plate-fin type is one of the most promising technologies due to its many benefits including simplicity, low cost, no cross contamination between airstreams, no moving parts, and high efficiency [8], [9]–10]. Generally, air-to-air plate-fin heat/energy recovery systems consist of corrugated fins placed between flat plates. The structure is brazed together forming a monolithic block. Without mixing with each other, two airstreams flow along the space between adjacent plates. However, the heat or/and humidity is harvested and transferred between airflows [4,10]. As referred by [1,^4^,^7^,^10^–[11], [12], the recovery potential of these air-to-air exchangers can reach up to 94% and 83% of sensible heat and latent heat, respectively. In order to recover more energy, new heat/energy exchangers designs (including core geometries, flow arrangements, materials, etc.) are brought to light and have ceaselessly evolved. In this context, important efforts have been, over the recent years, dedicated to evaluating and enhancing HRVs and ERVs performances [8–12]. Experimental investigations have been conducted [1,^4^,^8^,12] and many researchers have been interested in mathematical modeling using numerical calculation approaches (the effectiveness-NTU (known as ε−NTU) methods, computational fluid dynamics (CFD), and finite element methods (FEM)), and mainstream simulation platforms as well. In this respect, air-to-air heat/energy exchangers models are extensively developed in the literature to deeply evaluate the systems performances.

In [13], a pioneer modeling method was conducted by Wetter using the ε−NTU approach. In his research, the author developed an explicit simulation plate-type heat exchangers model to only evaluate the sensible heat transfer efficiency. The research findings indicated that this adopted method ensured a numerical stability coupled with a short computational time. Its main limitation is the use of an empirical correlation relying on a fixed air velocity exponent so as to evaluate the convective heat transfer coefficient [10,14].

Under unsteady flow conditions, Nakonieczny [15] suggested a numerical model of a plate-fin type heat exchanger in which detailed geometric parameters are heavily needed [10]. Furthermore, in order to discretize the unsteady-flow equations, a semi-discrete finite element method was adopted in this study. As consequence, the obtained results revealed that the proposed approach is computationally intensive and subsequently can cause convergence issues [10].

Thanks to Rose et al. [16] and Nielsen et al. [17], quasi-steady-state as well as dynamic models of counter-flow heat exchangers are put forward, respectively using discretization with finite element method (FEM). Apart from the resort to geometric data to evaluate the Reynolds number, the authors assessed, in their models, the dehumidification and frost formation impacts.

In [18], a model for a membrane-based energy recovery ventilator (MERV) with quasi-counter flow configurations was proposed using FLUENT CFD software. Based on humidity, temperature, and pressure non-dimensional values, Zhang considered the resistance of membrane moisture transfer as constant in this developed model. Furthermore, the author intentionally changed the Prandtl number and thermal diffusion values to be similar to Schmidt number and mass diffusion ones, respectively. Consequently, the conjugate problem of mass transfer was converted into a conjugate one of heat transfer.

Another detailed numerical analysis of HRV and MERV with counter/parallel flow arrangements has been carried out by Yaïci et al. [6], under typical winter and summer Canadian weathers. Relying on several assumptions, the authors developed a two-dimensional CFD model which was then validated with data gathered from the literature. Once validated, the proposed model was applied to assess the effect of numerous parameters on the system performances regarding sensible and latent efficiencies. As results, the authors stressed that the CFD approach has been proved to be a practical yet an effective design tool of air-to-air heat/energy recovery systems [6].

Similar investigations about three-dimensional CFD model of MERV were performed by Al-Waked et al. [19,20]. To validate the developed model, the authors resorted to published experimental works. Obtained results from validation pointed out that the proposed CFD model has been reasonably accurate in predicting the MERV performance.

To overcome the limitations of some existing models of air-to-air plate-fin heat exchangers, Zhou et al. [10] shed light on a new mathematical model based on the ε−NTU approach. Using a set of explicit equations, the recommended model was dedicated to calculating the heat transfer and flow resistance as well. Moreover, the authors highlighted that their model could be applied for various types of air-to-air heat exchangers without taking into account the dehumidification and if only some correlations must respect specific forms [10]. Despite its conspicuously short computational time compared to other models and the no requirement to detailed geometric information of exchanger, the proposed model was intended to be used for dry climatic operating conditions in which it can be only predict sensible heat effectiveness.

More recently, to predict the MERV performances regarding the energy exchange effectiveness, Qiu et al. [14] proposed a new approach which is based on two models: the cross-flow MERV efficiency numerical calculation model relying on the ε−NTU method and the multivariate polynomial regression model. A comparative analysis with existing numerical calculation methods revealed that the suggested approach did not need detailed core geometric data, was computationally more efficient and could be a promise tool in several engineering applications.

As for the use of mainstream simulation platforms, a wide range of research study has been a spate of interest in modeling air-to-air heat/energy recovery systems using TESS Library [21], TRNSYS 17 Library [22], Modelica Library [23], and so on. Nevertheless, using a fixed heat transfer coefficient, using a constant heat efficiency without taking into consideration the effects of changing conditions on heat transfer, etc. still represent their main limitations [10].

Modeling a single core HRVs and ERVs was extensively tackled in previous works as well as the adopted methods to evaluate their performances. In the current study, a modeling approach for a dual-core exchanger was proposed and an experimental validation was presented. In this regard, an appropriate and an accurate mathematical model for a hybrid dual-core exchanger (ERV-HRV in cascade connection), known as Hybrid 120, was developed in order to evaluate its energy recovery potential. Relying on the ε−NTU approach, a heat and mass transfer models are clearly presented for a thorough evaluation. Finally, the developed model validation was performed through a comparison between simulation results and real data gathered from laboratory environment experiments.

## Mathematical model development

**Fig. 1:**
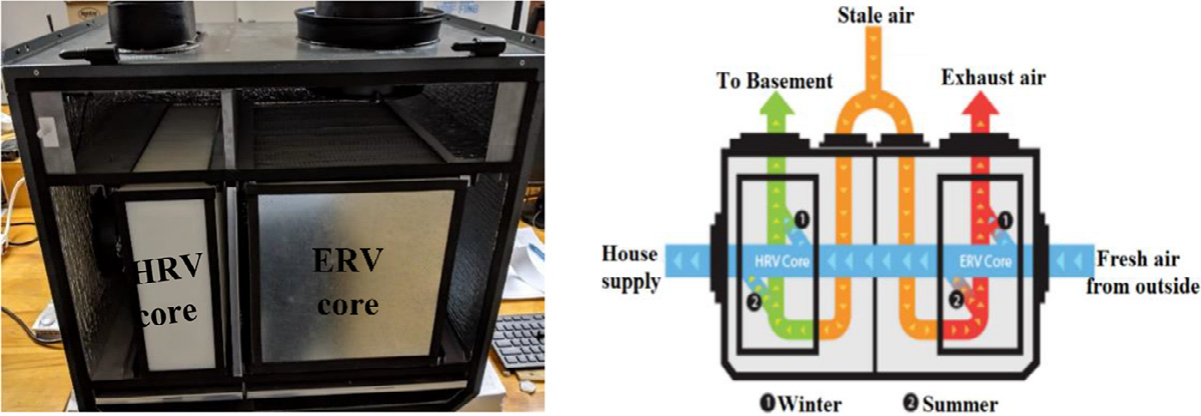
Hybrid dual-core system (ERV-HRV).

Manufactured by the ClairiTech Innovations company, the air-to-air total heat exchanger used in this study consists of a hybrid dual-core (ERV-HRV), three filters and three fans. Installed in cascade connection (Fig. 1), both the ERV and HRV cores have cross-flow plate-fin structures. Apart from the indoor air quality (IAQ) improvement and the moisture control, this ventilation system, which was dedicated to residential sector applications, recovers latent and sensible heat.

As depicted in Fig. 1, the indoor air along with toxins are continually exhausted to outdoor via the ERV core. While, the HRV core, which was reserved only to crawlspaces or/and basements, serves to raise the downstairs temperature by recirculating the warm air from the upstairs. Further, the fresh air was simultaneously treated twice through the ERV and HRV cores before its injection to the habitation.

As aforementioned, the intended investigation aims to develop an adequate model of the combined ventilation system illustrated in Fig. 1. To this end, two mathematical heat and mass transfer models are clearly presented relying on the ε−NTU method.

## Sensible and latent efficiencies determination

Commonly, three indicators are used to specify the total energy recovery potentials of the dual-core exchanger: Sensible efficiency $\varepsilon_{s}$, latent efficiency $\varepsilon_{l}$, and enthalpy efficiency $\varepsilon_{h}$ known as total exchange efficiency [5,^10^,14]. Mathematically speaking, these indicators are respectively given as:

(1) $$\varepsilon_{s}=\frac{{\dot{Q}}_{s}}{{\dot{Q}}_{s\_max}}$$

(2) $$\varepsilon_{L}=\frac{{\dot{Q}}_{L}}{{\dot{Q}}_{L\_max}}$$

(3) $$\varepsilon_{T}=\frac{{\dot{Q}}_{T}}{{\dot{Q}}_{T\_max}}$$

Where: ${\dot{Q}}_{s}$,${\dot{Q}}_{L}$ and ${\dot{Q}}_{T}$ are respectively the actual rates of sensible, latent and enthalpy heat transfer which are represented by [5,^6^,14]:

(4) $${\dot{Q}}_{s}={\dot{m}}_{1}c_{p1}|T_{1,out}-T_{1,in}\left|={\dot{m}}_{2}c_{p2}|T_{2,in}-T_{2,out}\right|$$

(5) $${\dot{Q}}_{L}={\dot{m}}_{1}l_{fg1}|w_{1,out}-w_{1,in}\left|={\dot{m}}_{2}l_{fg2}|w_{2,in}-w_{2,out}\right|$$

(6) $${\dot{Q}}_{T}={\dot{Q}}_{s}+{\dot{Q}}_{L}={\dot{m}}_{1}|h_{1,out}-h_{1,in}\left|={\dot{m}}_{2}|h_{2,in}-h_{2,out}\right|$$

Where: $T_{1,in},\;T_{1,out}$ represent the inlet and outlet temperatures of incoming fresh air, respectively;$\;w_{1,in},w_{1,out}$ are the inlet and outlet humidity ratios of incoming fresh air, respectively; $h_{1,in},\;h_{1,out}$ represent the inlet and outlet specific enthalpies of incoming fresh air respectively; $T_{2,in}$ $T_{2,out}$ represent the temperatures of stale air and exhaust air respectively; $w_{2,in}$ $w_{2,out}$ represent the humidity ratios of the stale air and exhaust air respectively; $h_{2,in},\;h_{2,out}$ are respectively the specific enthalpies of the stale air and exhaust air; ${\dot{m}}_{1}$,${\dot{m}}_{2}$ represent the mass flow rates of incoming fresh air and exhaust air respectively; $c_{p1}$,$c_{p2}$ are the specific heat capacities of inlet and outlet fresh air respectively; and $l_{fg}$ is the water vaporization's specific latent heat.

Further, the maximum possible rates of sensible, latent and enthalpy heat transfer can be respectively expressed by Eqs. (7)–(9) [6,10]:

(7) $${\dot{Q}}_{s\_max}={\dot{C}}_{min}\left|T_{2,in}-T_{1,in}\right|$$

(8) $${\dot{Q}}_{L\_max}={\dot{G}}_{min}\left|w_{2,in}-w_{1,in}\right|$$

(9) $${\dot{Q}}_{T\_max}={\dot{m}}_{min}\left|h_{2,in}-h_{1,in}\right|$$

Where: ${\dot{m}}_{min},$ ${\dot{G}}_{min}$ and ${\dot{C}}_{min}$ represent the minimum value of mass flow, latent flow, and thermal capacity flow rates between exhaust and fresh airstreams which are respectively given as:

(10) $${\dot{m}}_{min}=min\left\{{\dot{m}}_{1},{\dot{m}}_{2}\right\}$$

(11) $${\dot{G}}_{min}=min\left\{{\dot{m}}_{1}l_{fg1},{\dot{m}}_{2}l_{fg2}\right\}$$

(12) $${\dot{C}}_{min}=min\left\{{\dot{m}}_{1}c_{p1},{\dot{m}}_{2}c_{p2}\right\}$$

Moreover, to calculate the humid air's specific enthalpy, Eq. (7) was used [5]:

(13) $$h_{i,in/out}=c_{pi}T_{i,in/out}+\left(2501+c_{pm}T_{i,in/out}\right)\;w_{i,in/out}$$

Where: $C_{pm}$ represents the specific heat capacity at a constant pressure.

By substituting Eqs. (4) and (7) into Eq (1), the sensible heat recovery efficiency $\varepsilon_{s}$ can be written as:

(14) $$\varepsilon_{s}=\frac{{\dot{m}}_{1}c_{p1}\left|T_{1,in}-T_{1,out}\right|}{{\dot{C}}_{min}\left|T_{1,in}-T_{2,in}\right|}$$

Similarly, the latent heat recovery efficiency $\varepsilon_{l}$ can be expressed by rearranging Eqs. (5), (8) and (2):

(15) $$\varepsilon_{L}=\frac{{\dot{m}}_{1}l_{fg1}\left|w_{1,in}-w_{1,out}\right|}{{\dot{G}}_{min}\left|w_{1,in}-w_{2,in}\right|}$$

As for the total heat recovery efficiency $\varepsilon_{T}$, it can be given by substituting Eqs. (6) and (9) into Eq (3):

(16) $$\varepsilon_{T}=\frac{{\dot{m}}_{1}\left|h_{1,in}-h_{1,out}\right|}{{\dot{m}}_{min}\left|h_{1,in}-h_{2,in}\right|}$$

For balanced airflows, the sensible, latent and enthalpy efficiencies can be written respectively as follows [5,14]:

(17) $$\varepsilon_{s}=\frac{\left|T_{1,in}-T_{1,out}\right|}{\left|T_{1,in}-T_{2,in}\right|}$$

(18) $$\varepsilon_{L}=\frac{\left|w_{1,in}-w_{1,out}\right|}{\left|w_{1,in}-w_{2,in}\right|}$$

(19) $$\varepsilon_{T}=\frac{\left|h_{1,in}-h_{1,out}\right|}{\left|h_{1,in}-h_{2,in}\right|}$$

As reported by [5,14], the enthalpy efficiency can be further given by combining Eqs. (13) and (19):

(20) $$\varepsilon_{T}=C_{S}\times \varepsilon_{S}+C_{L}\times \varepsilon_{L}$$

Where: $C_{S}$ and $C_{L}$ referred to as weighted coefficients which expressed respectively by Eqs. (21)–(22):

(21) $$C_{S}=\frac{A}{A+B}$$

(22) $$C_{L}=\frac{B}{A+B}$$

Where: $A=\frac{1.005}{\max\left(w_{1,in},w_{2,in}\right)-min\left(w_{1,in},w_{2,in}\right)}$ and $B=\frac{2501}{\max\left(T_{1,in},T_{2,in}\right)-min\left(T_{1,in},T_{2,in}\right)}$

## Heat and mass transfer evaluation

To theoretically evaluate and predict the dual-core exchanger performances, simulations of both sensible and latent heat exchange in the combined system are crucial. To this end, two mathematical models of heat and mass transfer are then presented based on ε−NTU approach.

In the present study, some assumptions are considered as following: (1) No heat loss and no airflows leakage are considered, (2) The fouling and the material's thermal resistance are neglected; (3) The air properties are approximately evaluated; (4) The membrane resistance for heat conduction is neglected.

### Heat transfer model

By referring to the ε−NTU approach adopted by Zhou et al. [10], the general form of sensible heat exchange efficiency of the unmixed cross-flow dual-core ventilation system can be represented by Eq. (23) [7,10]:

(23) $$\varepsilon_{s}=1-exp\left\{\frac{NT{U_{s}}^{0.22}}{R_{c,s}}\left[exp\left(-R_{c,s}NT{U_{s}}^{0.78}\right)-1\right]\right\}$$

Where: NTU and $R_{c}$ represent the number of heat transfer units and capacity rate ratio, respectively. These dimensionless parameters are given as [7,10]:

(24) $$NTU_{s}=\frac{AU_{s}}{{\dot{C}}_{min}}$$

(25) $$R_{c,s}=\frac{{\dot{C}}_{min}}{{\dot{C}}_{max}}$$

Where: $U_{s}$ and A are the overall heat transfer coefficient and total surface of heat exchange, respectively; and ${\dot{C}}_{max}$ represents the maximum value of thermal capacity flow rate between exhaust and fresh airstreams which is expressed as follows:

(26) $${\dot{C}}_{max}=max\left\{{\dot{m}}_{1}c_{p1},{\dot{m}}_{2}c_{p2}\right\}$$

As for the overall heat transfer coefficient $U_{s}$, its general form is represented by [14,25]:

(27) $$U_{s}^{-1}=\frac{1}{h_{s}}+\frac{\delta }{\lambda_{m}}+\frac{1}{f_{s}}+\frac{1}{f_{e}}+\frac{1}{h_{e}}$$

Where: $h_{e}$ and $h_{s}$ represent the convective heat transfer coefficients of exhaust and supply airstreams, respectively; $f_{e}$ and $f_{s}$ are the fouling coefficients of exhaust and supply airflows, respectively; and δ and $\lambda_{m}$ are the core membrane thickness and thermal conductivity, respectively.

By neglecting the membrane resistance for heat conduction, the overall heat transfer coefficient can be further expressed by Eq (28):

(28) $$U_{s}^{-1}=\frac{1}{h_{s}}+\frac{1}{h_{e}}$$

Commonly, the evaluation of convective heat transfer coefficients of exhaust and supply airstreams depends on the calculation of several parameters. In this respect, a set of mathematical correlations including, Reynolds, Prandtl and Nusselt numbers are extensively highlighted based on simulations and experiments [3,^10^,25].

Regarding the Reynolds number, it is expressed by Eq. (29). While Eq (30) represents the Prandtl number:

(29) $$Re_{i}=\frac{V_{i}D_{h}\rho_{air,i}}{\mu_{air,i}}$$

(30) $$Pr_{i}=\frac{C_{p,i}\mu_{air,i}}{\lambda_{air,i}}$$

Where: i is an index which denotes the exhaust and supply airflows (e: exhaust and s: supply); $\mu_{air}$,$\rho_{air}$, and $\lambda_{air}$ represent the, dynamic viscosity, density and thermal conductivity of the air, respectively; V represents the velocity of air flow, and $D_{h}$ is the hydraulic diameter which is expressed as follows [3,10]:

(31) $$D_{h}=\frac{4S}{P}=\frac{2\;\times a\times L}{a+L}\approx 2\;\times a$$

Where: S and P are the cross-sectional surface and wetted perimeter, respectively; L and a are respectively the core width and space between adjacent plates.

Moreover, in order to calculate the Nusselt number, the correlation referred by Zhou et al. [10] was adopted in this investigation. To this end, the heat transfer Colburn factor correlation is given as:

(32) $$j=c_{1}c_{2}Re^{m}$$

Where: $c_{1}$ is a constant real number, $c_{2}$ represents a constant which absolutely depends on the geometry of the heat/energy cores, and m is the Reynolds number's exponent.

The above relation may be further expressed as [10]:

(33) $$j=\frac{Nu}{RePr^{1/3}}$$

Therefore, the Nusselt number was calculated by Eq. (34) which lead subsequently to calculate the convective heat transfer coefficients using Eq. (35) [10]:

(34) $$Nu=c_{1}c_{2}Re^{m+1}Pr^{1/3}$$

(35) $$h_{i}=Nu_{i}\frac{\lambda_{air,i}}{D_{h}}$$

Once the above parameters are calculated, the overall heat transfer coefficient $U_{s}$ as well as the sensible heat efficiency $\varepsilon_{s}$ are respectively calculated using Eqs. (28) and (23).

Hence, the temperatures of supply and exhaust airstreams can be predicted based on Eq. (36) and Eq. (37), respectively [10]:

(36) $$T_{1,out}=T_{1,in}+\varepsilon_{s}\frac{{\dot{C}}_{min}}{{\dot{m}}_{1}c_{p1}}\left(T_{2,in}-T_{1,in}\right)$$

(37) $$T_{2,out}=T_{2,in}-\frac{{\dot{Q}}_{s}}{{\dot{m}}_{2}c_{p2}}$$

### Mass transfer model

On the other hand, a mass transfer model is equally important to be developed to properly assess the total heat recovery potentials of the system. In this respect, the present study referred to the heat and mass transfer analogy.

According to the adopted ε−NTU approach, the latent heat efficiency can be written as:

(38) $$\varepsilon_{l}=1-exp\left\{\frac{NT{U_{l}}^{0.22}}{R_{c,l}}\left[exp\left(-R_{c,l}NT{U_{l}}^{0.78}\right)-1\right]\right\}$$

(39) $$NTU_{l}=\frac{AU_{l}}{{\dot{m}}_{min}}$$

(40) $$R_{c,l}=\frac{{\dot{m}}_{min}}{{\dot{m}}_{max}}$$

Where: ${\dot{m}}_{max}$ represents the maximum value of mass flow rates between exhaust and supply airstreams.

(41) $${\dot{m}}_{max}=max\left\{{\dot{m}}_{1},{\dot{m}}_{2}\right\}$$

Analogously to heat exchange, the overall moisture transfer coefficient $U_{l}$ can be represented as follows [3], [7], [12], [24], [25]:

(42) $$U_{l}^{-1}=\frac{1}{k_{mass,s}}+r_{mm}+\frac{1}{k_{mass,e}}$$

Where: $k_{mass,e}$ and $k_{mass,s}$ represent the convective mass transfer coefficients of exhaust and supply airstreams, respectively; and $r_{mm}$ is the plate's moisture resistance.

As pointed out by research studies [3,^7^,25], the calculation of this resistance is rather complicated, and it strongly depends on the membrane properties and operating conditions as well. In this investigation, the plate's moisture resistance calculation was performed based on a mathematical relation adopted by [3] which is given as:

(43) $$r_{mm}=\frac{\delta }{D_{wp}}$$

Where: $D_{wp}$ represents the mass diffusivity.

Regarding the convective mass transfer coefficients calculation, it is generally carried out using Sherwood correlations. Relying on the Chilton-Colburn analogy, the Sherwood number is given as [7,^12^,25]:

(44) $$Sh_{i}=Nu_{i}.Le^{-1/3}$$

Where: Le denotes the Lewis number which varies from 1.19 to 1.22 under atmospheric conditions [7,25].

The above formula may be further written as [5,^12^,25]:

(45) $$Sh_{i}=\frac{k_{mass,i}D_{h}}{D_{va}}$$

Where: $D_{va}$ represents the vapor diffusivity in the air.

Furthermore, the convective mass transfer coefficients can be expressed by rearranging Eqs. (35), (45) and (44):

(46) $$k_{mass,i}=\frac{h_{i}}{C_{pi}}Le^{-1/3}$$

It is obvious that the convective mass transfer coefficients calculation leads to the calculation of the overall moisture transfer coefficient $U_{l}$ (Eq. (42)), and latent heat efficiency $\varepsilon_{l}$ as well (Eq. (38)).

Finally, the humidity ratios of supply air and exhaust air can be predicted based on Eqs. (47) and (48), respectively:

(47) $$w_{1,out}=w_{1,in}+\varepsilon_{l}\frac{{\dot{G}}_{min}}{{\dot{m}}_{1}l_{fg1}}\left(w_{2,in}-w_{1,in}\right)$$

(48) $$w_{2,out}=w_{2,in}-\frac{{\dot{Q}}_{l}}{{\dot{m}}_{2}l_{fg2}}$$

## Method validation

The suitability of the developed model in predicting the combined system recovery performances was furthered, in this present study, by its validation against real data gathered from laboratory environment experiments. In this respect, the simulation results of sensible and latent heat efficiencies are compared with experimental measurements.

It is worthy to note that the dual-core exchanger was set up and its operating modes are monitored over three weeks in March 2019 in a laboratory environment at the University of Moncton. As for the model validation, collected measured data for March 7, 2019 are used. The real outside temperature was varied between −10°C and −18.6°C and the relative humidity range was between 40% and 77%, during this day. The combined ERV-HRV system was operated on low-speed mode where the air flow rates remained at 43.64 - 46.64 L/s and are closely balanced.

**Fig. 2:**
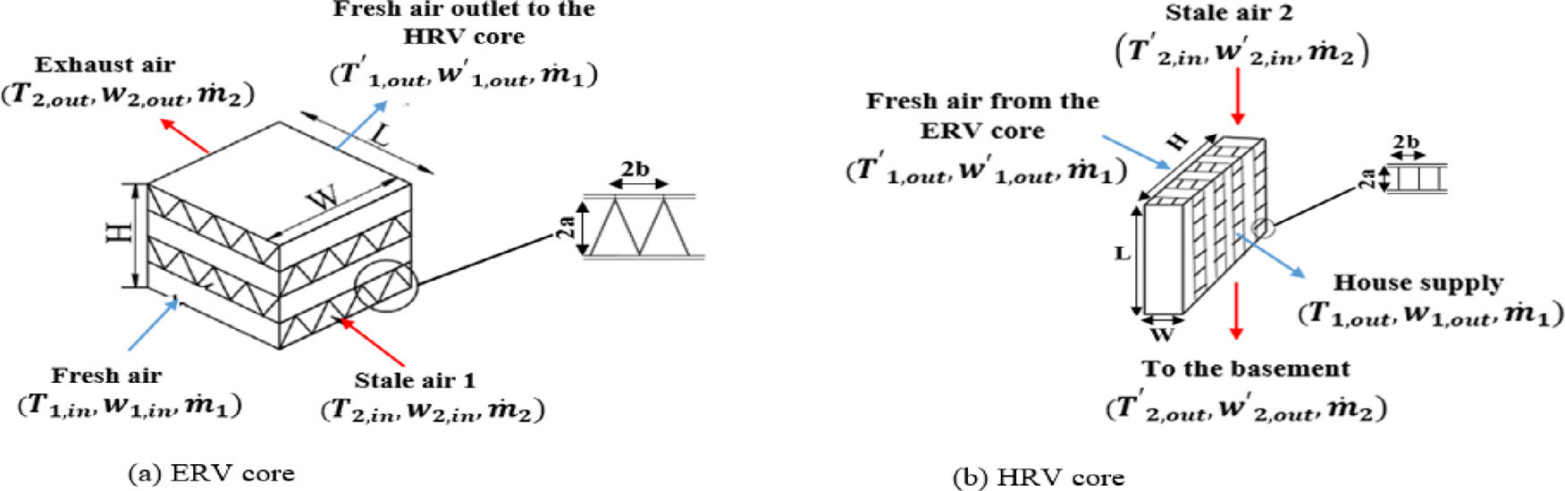
Cross-flow ERV and HRV structures.

The hybrid dual-core system geometry characteristics used to perform simulations are presented in Fig. 2 and Table 1.

**Table 1:** Combine ERV-HRV system geometrical specifications.

Parameters	ERV core	HRV core
Channels number for each flow: N	46	46
Duct height: 2a	3.8 mm	3.8 mm
Duct width: 2b	5 mm	5 mm
Core width: W	240 mm	94 mm
Core length: L	240 mm	240 mm
Core height: H	315 mm	340 mm

**Fig. 3:**
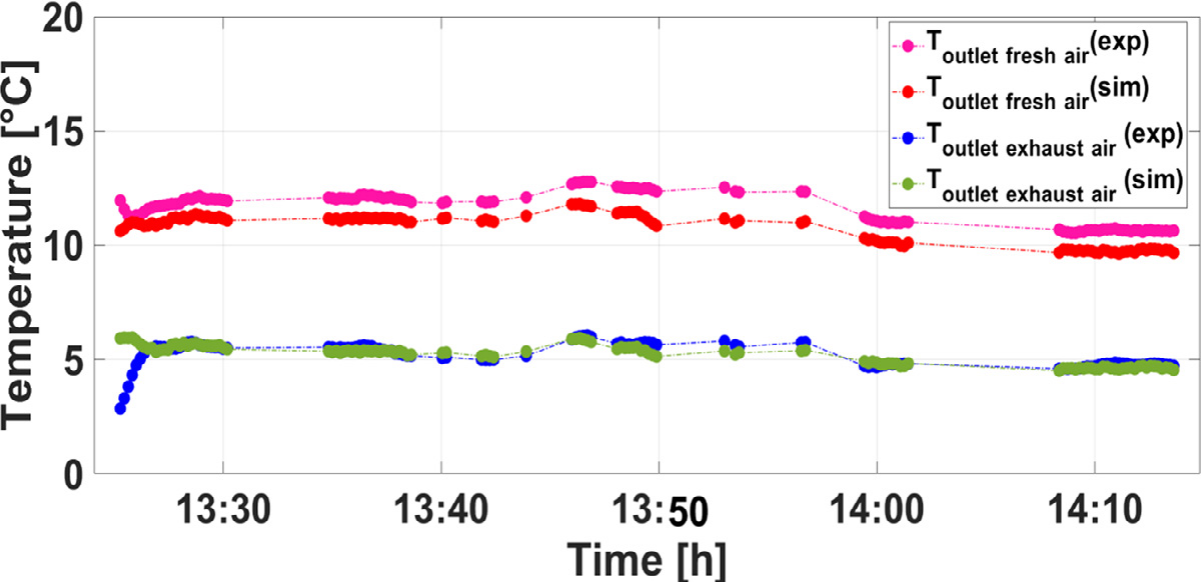
Comparison between simulation and experimental results of supply air and exhaust air temperatures.

**Fig. 4:**
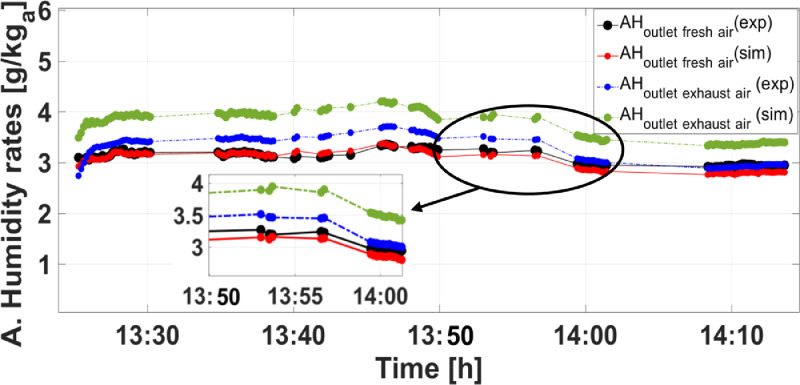
Comparison between simulation and experimental results of supply air and exhaust air humidity ratios.

**Fig. 5:**
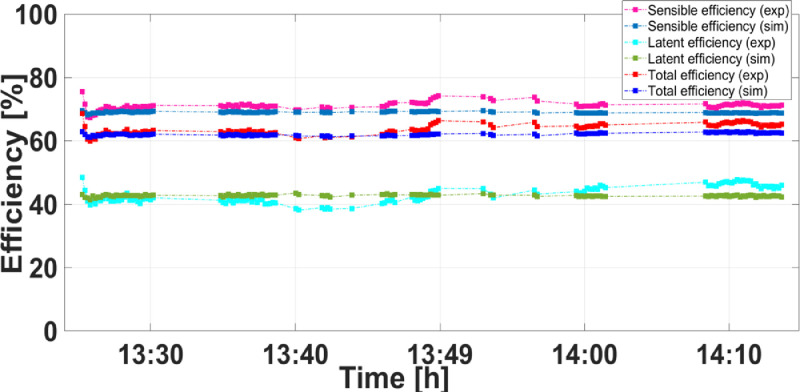
Comparison between simulation and experimental results of sensible, latent and total heat efficiencies.

So as to clearly validate the developed model, the temperatures and air humidity ratios of supply and exhaust airstreams are predicted for a set of given variable inputs. Figs. 3–5 show the comparison between results obtained from simulation and experiment in which the consistency between them is, generally, hard to be ensured. Commonly, owing to systematic as well as random errors of equipment used in experiments to measure and collect data which are subsequently used as inputs to validate the developed mathematical model.

As a rough rule of thumb, a discrepancy between simulation and experimental results was always obtained.

In this investigation, results agreement between measurements and simulation results was evaluated based on a relative discrepancy given as [10]:

(49) $$error=\frac{\left|Value_{exp}-Value_{sim}\right|}{Value_{exp}}\times \;100$$

Where: $Value_{sim}$ and $Value_{exp}$ represent results obtained from simulation and experiment, respectively.

Figs. 3 and 4 depict the comparison between theoretical and experimental results of supply air and exhaust air temperatures and humidity ratios, respectively.

As shown in Fig. 3, it is obvious that results obtained from simulations have a good agreement with experimental ones within maximum relative discrepancies of 9.9 % and 9.2 % for the supply air and exhaust air temperatures, respectively. Regarding the humidity ratios of supply and exhaust airstreams, reasonable agreements are noticed within maximum relative discrepancies of 5.67 % and 10.07 % between simulation and experimental results (Fig. 4).

As for the theoretical developed model predictions and experimental results of sensible, latent and total heat efficiencies, they are illustrated in Fig. 5. The mathematical model predicted that the maximum values of sensible, latent and total heat recovery efficiencies are around 70 %, 44 %, and 62 %, respectively. Under the same circumstance, the experimental results pointed out that the corresponding effectiveness are around 75.56%, 48.38 %, and 68.66 %. Therefore, reasonable agreements, within maximum relative discrepancies of 7.95%, 9.94 % and 8.53 %, are obviously observed.

## Conclusion

In this paper, an air-to-air dual-core exchanger was analyzed for a thorough prediction and assessment of its performance. To this end, a detailed mathematical model including heat and mass exchange mechanisms was clearly developed using the ε−NTU approach in order to carefully predict the ventilation system sensible and latent recovery efficiencies. Furthermore, the developed model was validated against real data to evaluate its suitability and accuracy. To sum up, the results disclosed that the developed mathematical model can be satisfactory for predicting the dual-core system performances in a conspicuously short time. A maximum relative discrepancy less than 10% was obtained between theoretical and experimental results which have a very good agreement. Therefore, the ε−NTU approach adopted in this study could be a convenient method for modeling air-to-air heat/energy recovery systems.

## Declaration of Competing Interest

The authors declare that they have no known competing financial interests or personal relationships that could have appeared to influence the work reported in this paper.
